# A new algorithm for an old problem: Reducing false alarms and alarm fatigue for ventricular tachycardia detection in the hospital setting

**DOI:** 10.1016/j.hroo.2023.10.003

**Published:** 2023-10-14

**Authors:** Margaret Harvey

**Affiliations:** Department of Acute and Tertiary Care, University of Tennessee Health Science Center, College of Nursing, Memphis, Tennessee

Continuous cardiac telemetry monitoring is the gold standard of care for detecting ventricular tachycardia (VT) in hospitalized patients. The most recent practice standards for electrocardiographic (ECG) monitoring in the hospital setting were updated in 2017,^1^ and they were largely based on the seminal work done by Drew et al,^2^ including ECG advances such as ST-segment and QTc monitoring. The updated practice guidelines address several concerns that unfortunately still exist today, such as the frequency of false alarms and alarm fatigue. False alarms may occur when electrodes are not properly placed on the patient, and estimates indicate 68%–99% of alarms are false or nonactionable.^1^ One common example is motion artifact, which may mimic VT and result in misdiagnosis and unnecessary interventions such as administration of antiarrhythmic drugs, diagnostic catheterization, and cardiac implantable electronic device implantation.^3^ Alarm fatigue occurs when nurses become desensitized due to the high frequency of false alarms, leading to action on nonactionable alerts such as unnecessary treatment, or missed true events such as lethal arrhythmias.^4^ The updated guidelines stress the need for proper skin preparation and lead placement, tailoring of alarm settings, and nursing education, noting the trend for continued overuse of ECG monitoring in patients when it is not indicated.^1^ Moreover, there is a need for research focusing on improved signal quality that reduces false or nonactionable alarms and increases specificity without an unacceptable loss of sensitivity.^5^

In a study reported in this issue of *Heart Rhythm O*^*2*^*,* in an effort to provide enhanced ECG signal quality and reduced false alarms for VT detection, Prasad et al^6^ sought to compare current bedside monitor VT alerts to VT alerts using a new algorithm developed by their research group and to explore the association between VT alerts and 30-day in-hospital mortality.^7^ A closed network system was used for ECG and physiological waveform data using GE Healthcare software, and data were sent to a secure and hospital-approved server maintained specifically for their research. To process new VTs, they used multichannel ECG waveforms, SpO_2_, and invasive arterial blood pressure (BP) waveforms. The new VT algorithm alerts are designed to decrease false VT by identifying factors such as motion/noise artifact, bundle branch block, and ventricular pacing as primary false sources of VT. Additional algorithm strategies include identifying P waves and correlating simultaneous drops in SpO_2_ and drops in invasive BP waveforms during a potential VT event. VT events identified using the new algorithm then were annotated as true or false based on a defined protocol conducted by PhD nurse scientists. Based on a total of 5670 intensive care unit (ICU) admissions, 503 (8.9%) experienced 30-day in-hospital mortality. VT events were higher in the current bedside monitor alert group (30%) compared to new-unannotated algorithm VT alerts (14.3%) and true-annotated VT (11.6%). Prasad et al also found that although there was no association of an increased rate of 30-day mortality with current bedside monitoring for VT (hazard ratio [HR] 1.06; 95% confidence interval [CI] 0.88–1.27), there was an increased association of 30-day hospital mortality using the new-unannotated algorithm (HR 1.38; 95% CI 1.12–1.69) and true-annotated VT (HR 1.39; 95% CI 1.12–1.73). Because 30-day hospital mortality was associated with unannotated and annotated true VT, Prasad et al conclude the new algorithm provides greater accuracy in identifying high-risk VT than current bedside monitoring for VT.

The work from Prasad et al^6^ highlights the ongoing issue of false alarms and alarm fatigue in the hospital setting, particularly in ICUs and stepdown units. The Joint Commission continues to call for alarm safety and issued a 2023 National Patient Safety Goal with recommendations to ensure that alarms on medical equipment are heard and that the response to those alarms are timely.^8^ Paine et al^9^ conducted a systematic review of physiological alarm characteristics and pragmatic interventions to reduce alarm frequency. Their search yielded 8 studies that evaluated interventions such as widening alarm parameters, instituting alarm delays, and using disposable ECG wires or frequently changing ECG electrodes. Although results are unproven and are at risk of bias, they conclude that the interventions are promising in reducing false alarms.^9^ Other available solutions to reduce alarm fatigue include monitoring only patients with indications, daily electrode changes and skin preparations, optimizing alarm state recognition, and continued education.^10^ Future solutions to improve algorithms include more sensitive/specific pattern recognition, simultaneous integration of multichannels, and inclusion of additional patient information.^10^

To that end, the new algorithm to detect VT reported in the study by Prasad et al^6^ is promising and integrates multichannel ECG waveforms and uses other physiological data such as simultaneous drops in invasive BP and SpO_2_. In addition, the study includes a large dataset with 5679 ICU admissions having 572,500 hours of continuous ECG monitoring over a timespan of 19 months. Although acknowledged limitations include the retrospective approach and the need for prospective studies, Prasad et al and their research team should be commended for their commitment to this important work and decades of research aimed at reducing false alarms and alarm fatigue. The new algorithm also holds promise for accurately identifying VT patients at greatest risk for mortality, and we look forward to further refinements addressing the sensitivity and specificity for detection of true VT. Scientists in the biomedical engineering community, such as Zhu et al,^11^ should also be encouraged to continue their work on novel methods to reduce noise and false alarms. False alarms and alarm fatigue continue to be an ongoing issue in the hospital setting, and there is a need for ongoing research, translation of current basic science, and data-driven interventions to ensure optimal patient safety.
